# Differences in force-time parameters and electromyographic characteristics of two high-velocity, low-amplitude spinal manipulations following one another in quick succession

**DOI:** 10.1186/s12998-020-00355-0

**Published:** 2020-12-08

**Authors:** Lindsay M. Gorrell, Philip J. Conway, Walter Herzog

**Affiliations:** 1 Human Performance Laboratory, Faculty of Kinesiology, University of Calgary, Calgary, Alberta Canada; 2 Chiropractor, Private Practice, Calgary, Alberta Canada; 3 Biomechanics Laboratory, School of Sports, Federal University of Santa Catarina, Florianopolis, SC Brazil

**Keywords:** SPINAL MANIPULATION, NECK PAIN, ELECTROMYOGRAPHY, NECK MANIPULATION

## Abstract

**Background:**

Spinal manipulative therapy is an effective treatment for neck pain. However, the mechanisms underlying its clinical efficacy are not fully understood. Previous studies have not systematically compared force-time parameters and electromyographic responses associated with spinal manipulation. In this study, force-time parameters and electromyographic characteristics associated with multiple manual high-velocity, low-amplitude cervical and upper thoracic spinal manipulations were investigated. The purpose of this analysis was to compare the force-time parameters and electromyographic characteristics between two spinal manipulations delivered following one another in quick succession if the first thrust was not associated with an audible cavitation.

**Methods:**

Nine asymptomatic and eighteen symptomatic participants received six Diversified-style spinal manipulations to the cervical and upper thoracic spines during data collected February 2018 to September 2019. Peak force, rate of force application and thrust duration were measured using a pressure pad. Bipolar surface electrodes were used to measure the electromyographic responses and reflex delay times in sixteen neck, back and limb outlet muscles bilaterally. Differences in force-time parameters and electromyographic data were analyzed between the first and second thrust.

**Results:**

Fifty-two spinal manipulations were included in this analysis. Peak force was greater (*p* < 0.001) and rate of force application faster (*p* < 0.001) in the second thrust. Furthermore, peak electromyographic responses were higher following the second thrust in asymptomatic (*p* < 0.001) and symptomatic (*p* < 0.001) subjects. Also, electromyographic delays were shorter in the symptomatic compared to the asymptomatic participants for the second thrust (*p* = 0.039). There were no adverse patient events.

**Conclusion:**

When a second manipulation was delivered because there was not audible cavitation during the first thrust, the second thrust was associated with greater treatment forces and faster thrust rates. Peak electromyographic responses were greater following the second thrust.

## Background

Spinal manipulative therapy is an effective nonpharmacologic treatment for neck and back pain [1–3]. Beneficial clinical changes, such as increased range of motion and decreased pain, are commonly reported following the delivery of spinal manipulation. However, the mechanisms underlying these beneficial treatment outcomes are not fully understood. One plausible explanation is that spinal manipulation causes a reflexive relaxation of muscles adjacent to the site of application and there is an expanding volume of literature investigating the relationship between spinal manipulation and electromyographic (reflex) responses. Specifically, several studies have investigated the relationship between force-time parameters (e.g. magnitude of peak force, preload force, duration of thrust) and the electromyographic (reflex) responses of musculature adjacent to the site of manipulation using both an animal model [4–9] and healthy adults [10–13] but, only one study has investigated this relationship in symptomatic patients [14].

To date, studies conducted on humans involved either a single thrust to a thoracic vertebra using a robot [10–12, 14], or thrusts applied to multiple levels of the spine utilizing different manual manipulation mechanics [15–18]. Specifically, Herzog et al. applied prone manual manipulations to C2 and C3 in asymptomatic participants [15]. Symons et al. delivered prone instrument (Activator®) manipulations to C2/C3 and T2/T3 asymptomatic participants. Similar to Herzog et al., other regions of the spine (thoracic, lumbar and lumbopelvic) were also manipulated [16]. In another study, Herzog et al. applied a prone reinforced hypothenar contact in a posterior to anterior direction to the left transverse processes of thoracic vertebra in two asymptomatic participants [17]. Finally, Suter et al. manipulated the sacroiliac joint of symptomatic participants [18]. In animal studies, lumbar manipulations were applied and electromyographic responses were measured using indwelling electrodes in anesthetized animals [4–6, 9, 19]. Collectively, these studies report that with higher peak forces, faster rates of force application, and shorter thrust durations, neural responses are generally increased.

Other studies have been conducted investigating the ability of experienced manual therapy clinicians to control the force-time parameters of high-velocity, low-amplitude (HVLA) spinal manipulation [20–22] during a single spinal manipulation delivered in a laboratory setting. However, to our best knowledge, this is the first study reporting on the differences between force-time parameters and associated electromyographic responses between two manual HVLA manipulations delivered by a clinician and following one another in quick succession.

Furthermore, despite uncertainty regarding the exact mechanism, many clinicians (and patients) judge the success of an HVLA manipulation by the presence of a cracking, clicking or popping sound – commonly known among manual therapists as cavitation [23–26]. Indeed, many practitioners will immediately deliver a second thrust in a clinical situation if cavitation is not achieved with the first thrust as it is anecdotally believed that cavitation is related to the successful delivery of HVLA manipulation [26, 27]. Despite this widespread assumption that cavitation is associated with the clinical benefits of spinal manipulation, there are conflicting reports in the literature as to the role of cavitation and reflex responses. Specifically, Brodeur et al. reported that cavitation ‘provides a simple means for initiating reflex actions’ [28] while other studies have reported that spinal manipulations not resulting in cavitation still elicited reflex responses [29, 30]. In these studies, the authors report that cavitation, when elicited by the slow application of force to facet joints, did not result in a reflex response and therefore, cavitation alone cannot be responsible for reflex responses. Thus, there is controversy as to the role of cavitation and reflex responses and this was addressed here.

The objective of this study was to compare the force-time parameters and electromyographic characteristics of manual HVLA cervical and upper thoracic spinal manipulations delivered following one another in quick succession when there was not audible cavitation with the first thrust in an asymptomatic and a symptomatic population. We hypothesized that there would be differences in the force-time parameters and electromyographic responses between the two thrusts, and that these responses would also differ between the asymptomatic and symptomatic populations.

## Methods

### Participants

This analysis is based on data collected in two studies, both of which were designed as descriptive observational investigations [13, 31]. Recruitment for these studies took place between February 2018 – September 2019, with participants aged 18 to 60 years responding to researcher’s call for volunteers. A convenience sampling method was used. In the first study, asymptomatic volunteers aged 18 to 40 years attending the University of Calgary were recruited. Volunteers attended an initial session and if all inclusion criteria were met, were subsequently scheduled to attend a data collection session occurring at the University not more than four days after the initial visit. In the second study, symptomatic individuals attending a private chiropractic clinic for treatment of mechanical neck pain were recruited. These volunteers attended a single combined screening and data collection session occurring at the clinic. All participants were screened for contraindications by the same registered, practicing chiropractor. Contraindications included a personal or family history of a connective tissue disorder, current use of anticoagulant therapy, history of recent surgery and/or neck trauma, facial or intra-oral anesthesia or paresthesia, visual disturbances, dizziness and/or vertigo. Furthermore, symptomatic volunteers were excluded if they presented with cervical or upper thoracic pain distribution which was not consistent with mechanical dysfunction or did not originate from the cervical and/or upper thoracic spines. In this instance, mechanical neck dysfunction was defined as pain in the cervical or occipital regions not associated with an identified pathological cause [3, 32].

For both cohorts, following screening, if no contraindications to cervical and upper thoracic spinal manipulation were present, a targeted medical history and physical examination were performed by this same chiropractor. In accordance with the current literature and clinical practice guidelines, vertebral artery safety tests were not performed [33–35]. All symptomatic participants completed a Neck Disability Index prior to enrollment. This outcome measure, which correlates higher scores with greater disability, has been validated as reliable in patients with mechanical neck pain [36, 37]. Once the chiropractor was confident that there were no contraindications and that the volunteer met all inclusion criteria, participants were enrolled into the study.

All participants provided written, informed consent and all procedures were carried out in accordance with the Declaration of Helsinki and were approved by the University of Calgary Health Research Ethics Board (REB16–0296).

### Treatments

Symptomatic and asymptomatic participants received six Diversified-style, manual HVLA spinal manipulations to the cervical and upper thoracic spines. These manipulations were delivered in a set order – C1, C2, C6, C7, T1 and T4 by a second registered and practicing chiropractor with over 30 years' experience in the delivery of manual spinal manipulation. A one-off coin-flip determined that the right side was the first to be treated for all participants and each subsequent manipulation was alternated between the left and right sides with a two-minute rest period between each trial. A trial was defined as all manipulations applied to a vertebra i.e. C1 was a single trial, C2 was another etc. This rest period was provided to optimize participant comfort.

For all cervical spinal manipulations, the participant was positioned supine with the head supported by the clinician’s hands. The articular process of the involved vertebra was contacted by the antero-lateral aspect of the proximal phalanx of the second digit of the clinician’s index finger. The head was then taken into flexion, ipsilateral lateral flexion and contralateral rotation to the pre-manipulative position. A rapid, controlled low-amplitude thrust was applied in a further posterior-anterior line of drive to deliver the manipulation [38]. Ipsilateral in this instance means the same side as the contact i.e. for manipulation of C1, the right side of the participant’s neck was contacted, and rotation of the head occurred to the left.

For all upper thoracic manipulations, the participant was positioned prone on the treatment table. The transverse processes of the involved vertebra were contacted with a bilateral hypothenar-heel contact in which the hands were perpendicular to each other, specifically the fingers of the right hand faced supero-laterally (to the left shoulder) and the fingers of the left hand faced supero-laterally to the right shoulder. A transfer of the clinician’s weight was utilized in a posterior-anterior and inferior-superior direction to deliver the manipulation [38]. Throughout the study, and at the discretion of the treating clinician, if the first thrust was deemed unsuccessful (no audible cavitation), a second thrust was immediately applied as often occurs in clinical practice.

### Time of manipulation onset and force measurement

To ensure that the electromyographic responses were associated with the applied manipulation, the time of manipulation onset was recorded using a thin, flexible pressure pad. This pressure pad was placed between the clinician’s contact and the participant’s neck or thoracic spine [39]. The pad used was 2.2 mm thick and contained 99 sensors which detect pressures in the range of 20–600 kPa with a resolution of 2.5 kPa. Pressure data were collected at 200 Hz via Bluetooth® transmission and were converted to force data in the following way. The pressure values in all ‘non-zero’ cells were multiplied by the corresponding area of each sensor to obtain the force for each cell. The individual cell force values were then summed at each time point to provide the overall force. The two measurement systems were synchronized using a 5 V square wave pulse that was sent from the force measurement system (Pedar®, Novel, Munich, Germany) to the EMG data collection computer, so that a common reference time point was visible in both systems. The leading edge of this square pulse was defined as time zero for both measurement systems. Knowing the frequency of data collection for the force and EMG measurement systems (200 Hz and 999 Hz, respectively), the corresponding time interval between data points could be calculated (5 ms and 1.001 ms, respectively), and was used for synchronizing specific events of interest between the force and EMG systems*.*

### Electromyographic recordings

**Fig. 1:**
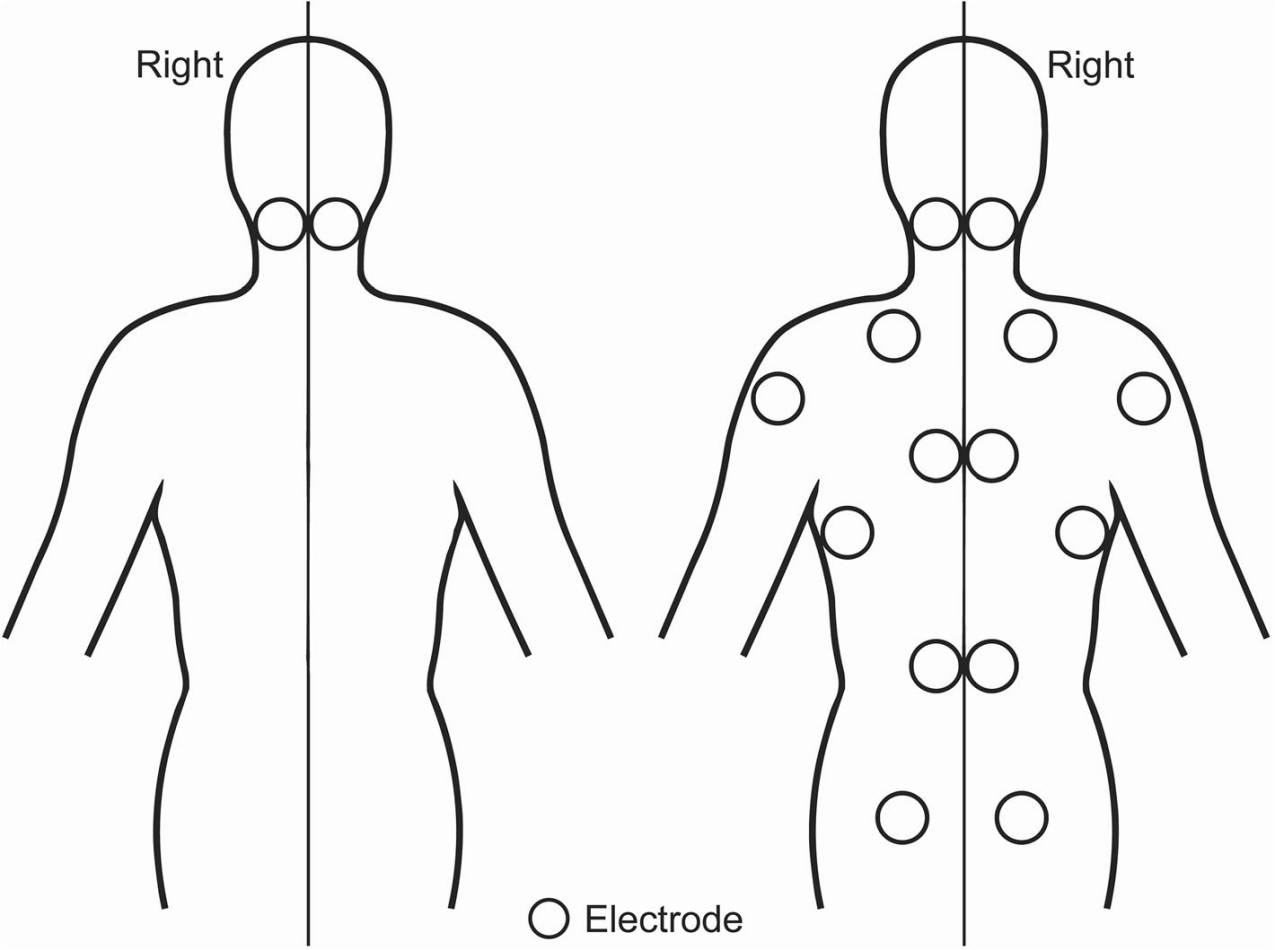
Schematic drawing of electromyographic electrode placement (left panel – anterior view & right panel – posterior view)

Electromyographic responses during manipulation were measured using bipolar surface electrodes. Prior to the placement of electrodes on each of the target areas, the skin was thoroughly cleansed using gauze soaked in a 70% ethanol solution and skin debridement was achieved using a disposable razor. Sixteen pairs of electrodes (SOFTRACE® Medium, ConMed Corporation, Utica, USA) were then carefully placed bilaterally on the following muscles: sternocleidomastoid, splenius cervicis, upper trapezius, posterior deltoid, middle trapezius, latissimus dorsi, longissimus thoracis and gluteus maximus (Fig. 1). These muscles were chosen as it has been reported that electromyographic responses associated with spinal manipulation are local and non-local [13, 15, 40]. Following electrode placement, ensuring an inter-electrode distance of 30 mm, conductance was tested using an AC impedance meter (Grass Instruments, RI, USA) and where necessary, the skin preparation and electrode placement process was repeated until all electrode impedance values were below 5 kΩ. Once the leads to the amplifiers (Biovision, Wehrheim, Germany) were attached, flexible tape (Fixomull® transparent) was applied over the electrodes to secure the leads in place to prevent movement artifact within the electromyographic recording. All care was taken by the clinician to avoid direct contact over both electrodes and leads during each trial and, trials in which this occurred were removed from the analysis. Cable-integrated amplifiers with a common-mode rejection ratio (CMMR) of 120 dB, and signal to noise rate of 1 μV were used (Biovision, Wehrheim, Germany). Individual electrode amplification gain (500-5000x) was checked, adjusted if necessary and recorded for every lead prior to each trial. A reference ground electrode was placed on the right lateral epicondyle of all participants. Electromyographic signals were collected at 999 Hz per channel via an analog-to-digital converter (Windaq, DATAQ Instruments Inc., Akron, USA). Data were stored on a personal laptop computer for off-line analysis.

### Data analysis

**Fig. 2:**
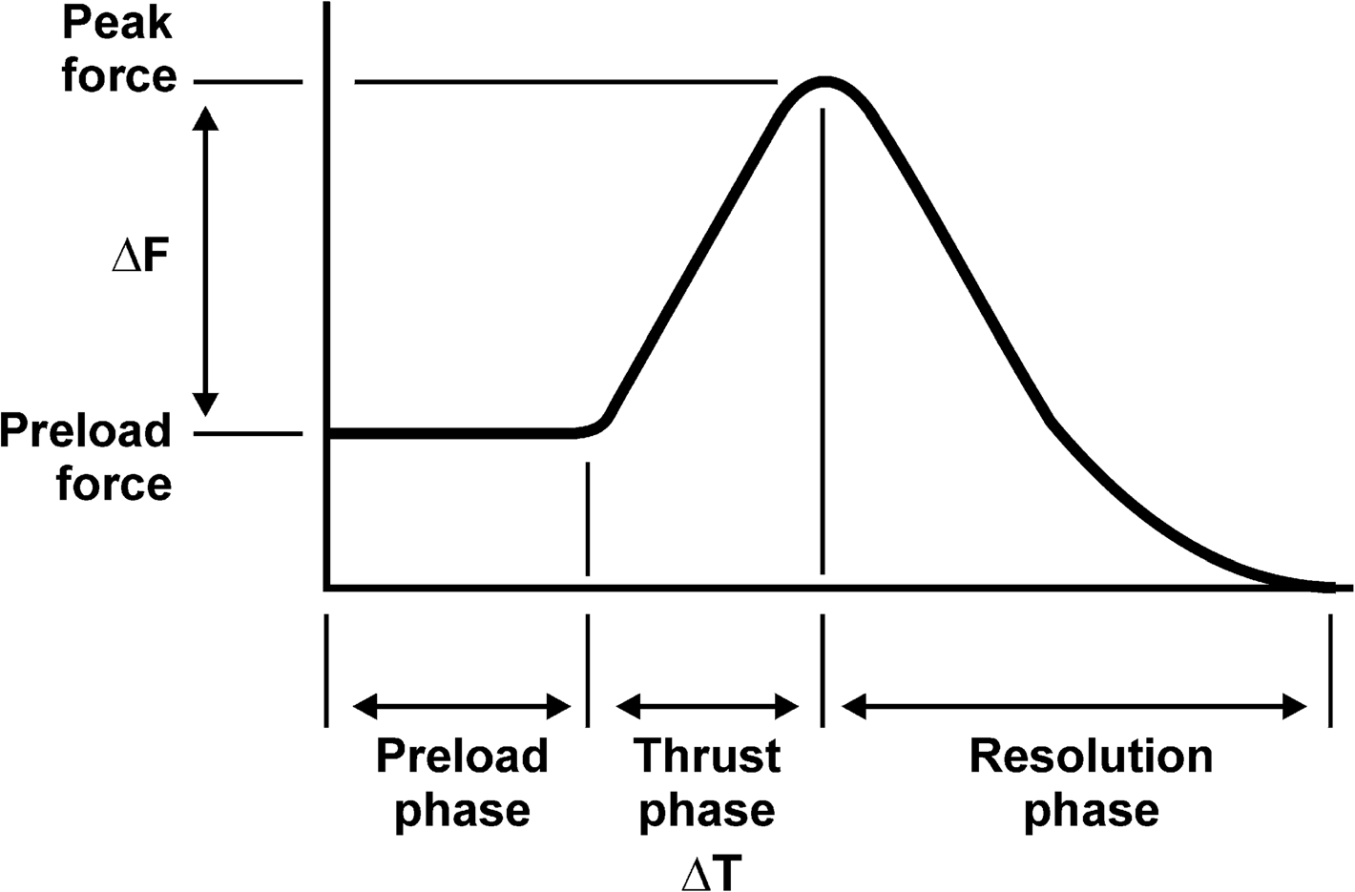
Typical force-time profile for spinal manipulation. Δ: change in, F: force, T: time

Data were exported for analysis using a custom-built computer script (Matlab Version R2019b; Mathworks, Natick, MA). Electromyographic data were filtered using a 3rd order, 150 Hz high-pass zero-phase Butterworth filter. The filter eliminated the contribution of heartbeat to the electromyographic signal. An electromyographic response was defined as an increase in the electromyographic signal of greater than 2 standard deviations above baseline and occurring within 250 ms of the onset of manipulation. Electromyographic responses were normalized to the first thrust in a pair of manipulations. The onset of manipulation was defined as the lowest force value of the downward incisural dip (at the end of the preload phase), according to the force-time profile (Fig. 2). Preload force was defined as the average force in the 500 ms preceding the onset of the thrust. In this analysis, only trials involving two thrusts, a first thrust (did not result in cavitation) followed immediately (within 2 s) by a second thrust, were analyzed. Trials involving a single thrust have been reported previously [13, 31]. There were no instances in which more than two thrusts were applied. In general, the second thrust did cavitate however there were a very small number of instances where cavitation did not occur with either thrust. It was decided to include these second thrusts that did not cavitate in the analysis as they displayed similar force-time parameters and electromyographic responses as the other second thrusts. Peak electromyographic response was defined as the maximum root-mean-square (RMS) value calculated using a sliding window (width = 50 ms; step size = 1 ms) for each of the channels during the interval between the thrust onset and 25 ms after peak force occurrence. The delay of onset of the electromyographic signal was defined as the period of time between the onset of the treatment thrust and the instant when the RMS of the electromyographic signal exceeded 2 standard deviations of the electromyographic baseline value. Force data were analyzed for peak force (highest force reached during manipulation), average rate of force application (calculated as the peak force minus the preload force divided by the duration of the thrust (Fig. 2)), and thrust duration (time of onset of the thrust to the time of peak force occurrence). Differences in thrust force-time parameters and electromyographic responses were calculated using Wilcoxson Signed Rank Testing Exact method. Between-group differences were calculated using Mann-Whitney U tests. These analyses were performed in the IBM® SPSS Statistics 26 program. Bonferroni corrections were applied to account for possible Type I errors due to multiple comparisons and were performed in Microsoft Excel (version Office 365, 2020). Statistical significance was set at *p* < 0.05.

## Results

Data from 27 participants (9 asymptomatic and 18 symptomatic) were used in this analysis, of which 48% were female. Participants were 21–48 years old (mean: 32 ± 9), had an average height of 171 cm (± 12 cm) and an average weight of 74 kg (± 20 kg) (Table 1). Symptomatic participants had an average Neck Disability Index of 6 (± 1) points (maximum score is 50 points). There were no differences between asymptomatic and symptomatic participants for: sex (*p* > 0.99), age (*p* = 0.082), height (*p* = 0.910) or weight (*p* = 0.250). Of the 232 manipulations delivered in the two studies, 52 (22%) were repeated as they were not associated with an audible cavitation in the first thrust and these were included in the analysis.

**Table 1:** Demographic information about the two cohorts

	Number of participants	Sex (% female)	Age (years)	Height (cm)	Weight (kg)
Asymptomatic	9	44	29 ± 4^#^	169 ± 8	65 ± 9
Symptomatic	18	50	33 ± 10	172 ± 14	79 ± 22

^#^ standard deviation

### Force-time parameters

Peak force was higher (*p* < 0.001) and rate of force application was faster (*p* < 0.001) in the second thrusts (Table 2). Peak force was higher for second thrusts delivered to the lower cervical spine (symptomatic: *p* < 0.001) and in the thoracic spine (asymptomatic: *p* = 0.004; and symptomatic: *p* < 0.001). Despite higher absolute peak force values for the cervical spine in the asymptomatic group and the thoracic spine in the symptomatic group, there were no statistically significant differences for the first thrust between the two groups (*p* = 0.439) or the second thrust between the two groups (*p* = 0.253). The rate of force application was faster in second thrusts delivered to the lower cervical spine in the symptomatic (*p* < 0.001) population and the thoracic spine in asymptomatic (*p* = 0.004) and symptomatic (*p* < 0.001) participants. The rate of force application was not different in symptomatic compared to asymptomatic participants for the first (*p* = 0.557) or second (*p* = 0.935) thrusts.

**Table 2:** Force-time parameters of cervical and upper thoracic spinal manipulations delivered following one another in quick succession in asymptomatic and symptomatic participants

		Peak force (*N*)		Rate of force application (N/s)		Thrust duration (ms)	
	Thrust	Asymptomatic	Symptomatic	Asymptomatic	Symptomatic	Asymptomatic	Symptomatic
Upper cervical spine	1	273^	136 ± 29^#^	1074^	601 ± 269	180^	93 ± 54
	2	407	174 ± 34	1787	895 ± 214	160	104 ± 32
	*p*-value						
Lower cervical spine	1	193 ± 74	174 ± 37	638 ± 98	912 ± 271	147 ± 12	103 ± 43
	2	248 ± 112	229 ± 62*	895 ± 328	1502 ± 731*	147 ± 12	112 ± 34
	*p*-value		< 0.001		< 0.001		
Thoracic spine	1	368 ± 71	490 ± 77	1732 ± 461	2749 ± 661	142 ± 19	108 ± 27
	2	464 ± 109*	573 ± 90*	2724 ± 587*	3710 ± 815*	120 ± 10	111 ± 6
	*p*-value	0.004	0.000	0.004	0.000		

* difference between thrusts significant (*p* < 0.05)

^ data only available from a single trial of two thrusts delivered to one participant

^#^ standard deviation

### Electromyographic characteristics

Peak electromyographic responses were greater following the second thrust in the asymptomatic (*p* < 0.001) and the symptomatic (*p* < 0.001) populations. Typically, there were more differences in the electromyographic responses between the two thrusts (i.e. a higher count) in the symptomatic compared to the asymptomatic population, both ipsilateral and contralateral to the side of contact for both neck (*p* = 0.008; *p* < 0.001) and back muscles (*p* < 0.001; *p* < 0.001) respectively. However, the magnitude of difference between thrusts was typically greater in the asymptomatic group (Table 3).

**Table 3:** Normalized differences in peak electromyographic responses associated with two cervical and upper thoracic spinal manipulations delivered following one another in quick succession in asymptomatic and symptomatic participants

		Left								Right							
		*Glut*	*Lats*	*Long*	*MidT*	*PDelt*	*SCM*	*Splen*	*UppT*	*Glut*	*Lats*	*Long*	*MidT*	*PDelt*	*SCM*	*Splen*	*UppT*
Asymptomatic	1	0.0082^+^± 0.0106^#^	0.0044 ± 0.0030	0.0334 ± 0.0437	0.0565 ± 0.0648	0.0198 ± 0.0314	0.1276 ± 0.1425	0.0550 ± 0.0328	0.0533 ± 0.0565	0.0088 ± 0.0107	0.0135 ± 0.0071	0.0442 ± 0.0606	0.0670 ± 0.0859	0.0199 ± 0.0227	0.1821 ± 0.2006	0.0547 ± 0.0315	0.0532 ± 0.0525
	2	0.0103 ± 0.0115	0.0054 ± 0.0035	0.0580 ± 0.0632	0.0753 ± 0.0852	0.0162 ± 0.0178	0.1399 ± 0.1783	0.0532 ± 0.0164	0.0433 ± 0.0539	0.0137 ± 0.0179	0.0176 ± 0.0117	0.0708 ± 0.0814	0.0699 ± 0.0709	0.0141 ± 0.0132	0.1752 ± 0.2355	0.0676 ± 0.0396	0.0732 ± 0.0734
	% diff	**25.10**	**20.99**	73.60* *p* = 0.002	**33.32**	**−17.89**	**9.61**	**−3.30**	**−18.73**	**55.08**	**30.60**	**60.16**	**4.28**	**−28.99**	**−3.81**	**23.60**	**37.57**
Symptomatic	1	0.0028 ± 0.0024	0.0096 ± 0.0090	0.0167 ± 0.0202	0.0138 ± 0.0159	0.0071 ± 0.0048	0.0452 ± 0.0419	0.0222 ± 0.0178	0.0342 ± 0.0819	0.0027 ± 0.0016	0.0108 ± 0.0122	0.0147 ± 0.0176	0.0206 ± 0.0365	0.0054 ± 0.0076	0.0295 ± 0.0252	0.0205 ± 0.0124	0.0183 ± 0.0298
	2	0.0031 ± 0.0022	0.0107 ± 0.0093	0.0243 ± 0.0301	0.0160 ± 0.0147	0.0084 ± 0.0079	0.0547 ± 0.0479	0.0275 ± 0.0246	0.0322 ± 0.0619	0.0031 ± 0.0023	0.0129 ± 0.0170	0.0182 ± 0.0218	0.0202 ± 0.0241	0.0057 ± 0.0057	0.0345 ± 0.0357	0.0236 ± 0.0148	0.0297 ± 0.0448
	% diff	**9.51**	**11.32**	45.87* *p* < 0.001	**15.78**	**17.96**	**20.79**	**23.96**	**−5.99**	16.27* *p* = 0.005	**20.03**	24.24* *p* < 0.001	**−2.19**	**6.46**	**17.27**	**15.11**	62.04* *p* < 0.001

*Glut* gluteus maximus, *Lats* latissimus dorsi, *Long* longissimus thoracis, *MidT* middle trapezius, *PDelt* posterior deltoid, *SCM* sternocleidomastoid, *Splen* splenius cervicis, *UppT* upper trapezius

^+^ raw value in mV

^#^ standard deviation

diff: normalized difference between thrust 1 and 2

* difference between thrusts significant (*p* < 0.05)

Asymptomatic participants had shorter electromyographic delays with the second thrust in neck (*p* = 0.001) and back (*p* < 0.001) muscles, while the corresponding values were shorter in the back muscles only for the symptomatic participants (*p* < 0.001) (Table 4). Electromyographic delays were shorter in the symptomatic compared to the asymptomatic participants for the first thrust (*p* = 0.039) but not the second (*p* = 0.061).

**Table 4:** Normalized differences in electromyographic delays associated with two cervical and upper thoracic spinal manipulations delivered following one another in quick succession in asymptomatic and symptomatic participants

		Left								Right							
		*Glut*	*Lats*	*Long*	*MidT*	*PDelt*	*SCM*	*Splen*	*UppT*	*Glut*	*Lats*	*Long*	*MidT*	*PDelt*	*SCM*	*Splen*	*UppT*
Asymptomatic	1	0.0836^+^ ± 0.0438^#^	0.0768 ± 0.0402	0.0501 ± 0.0291	0.0649 ± 0.0446	0.0724 ± 0.0283	0.0518 ± 0.0337	0.0475 ± 0.0290	0.0587 ± 0.0304	0.0852 ± 0.0407	0.0798 ± 0.0481	0.0582 ± 0.0278	0.0531 ± 0.0264	0.0723 ± 0.0238	0.0503 ± 0.0275	0.0484 ± 0.0234	0.0433 ± 0.0204
	2	0.0543 ± 0.0436	0.0581 ± 0.0380	0.0374 ± 0.0266	0.0281 ± 0.0163	0.0500 ± 0.0310	0.0388 ± 0.0340	0.0359 ± 0.0423	0.0392 ± 0.0183	0.0759 ± 0.0546	0.0472 ± 0.0249	0.0260 ± 0.0207	0.0438 ± 0.0319	0.0491 ± 0.0108	0.0409 ± 0.0375	0.0379 ± 0.0458	0.0287 ± 0.0173
	% diff	**−35.06**	**−24.32**	**−25.38**	**−56.75**	−30.92* *p* = 0.004	**−25.05**	**−24.49**	**−33.19**	**−10.82**	**−40.81**	−55.30* *p* < 0.001	**−17.53**	**−32.09**	**−18.82**	**−21.61**	**− 33.70**
Symptomatic	1	0.0445 ± 0.0288	0.0496 ± 0.0472	0.0239 ± 0.0173	0.0438 ± 0.0532	0.0372 ± 0.0271	0.0175 ± 0.0162	0.0272 ± 0.0221	0.0536 ± 0.0590	0.0577 ± 0.0355	0.0521 ± 0.0551	0.0228 ± 0.0185	0.0371 ± 0.0372	0.0386 ± 0.0261	0.0180 ± 0.0182	0.0283 ± 0.0281	0.0286 ± 0.0213
	2	0.0593 ± 0.0516	0.0338 ± 0.0258	0.0198 ± 0.0257	0.0363 ± 0.0413	0.0336 ± 0.0289	0.0210 ± 0.0270	0.0222 ± 0.0236	0.0311 ± 0.0304	0.0413 ± 0.0261	0.0448 ± 0.0514	0.0162 ± 0.0158	0.0317 ± 0.0408	0.0510 ± 0.0610	0.0223 ± 0.0289	0.0303 ± 0.0298	0.0339 ± 0.0522
	% diff	**33.15**	**−31.69**	**−17.08**	**−17.20**	**−9.74**	**20.51**	**−18.33**	**−41.91**	**−28.32**	**−14.06**	**−28.77**	**−14.54**	**32.04**	**23.71**	**6.97**	**18.69**

*Glut* gluteus maximus, *Lats* latissimus dorsi, *Long* longissimus thoracis, *MidT* middle trapezius, *PDelt* posterior deltoid, *SCM* sternocleidomastoid, *Splen* splenius cervicis, *UppT* upper trapezius

^+^ raw value in ms

^#^ standard deviation

diff: normalized difference between thrust 1 and 2

* difference between thrusts significant  (*p* < 0.05)

### Adverse events

No adverse patient events were reported in this study.

## Discussion

This is the first systematic analysis of force-time parameters and electromyographic characteristics associated with two manual HVLA manipulations delivered by a clinician and following one another in quick succession. Our finding that the second thrust occurred with higher peak force and faster rates of force application agrees with the literature that reports experienced clinicians are able to modify these force-time parameters to achieve a “successful” spinal manipulation [20–22]. Furthermore, our general finding of greater electromyographic response and shorter delay is also consistent with the literature which reports that with higher peak forces and faster rates of force application (as seen with our second thrusts), neural responses are generally increased [4–6, 9–19]. The congruence of our results with the published literature is encouraging, especially considering the significant methodological differences between our work and those used in previous studies (outlined earlier in this manuscript).

However, in contrast to our results, Currie et al. reported that participants who are experiencing low back pain (i.e., are symptomatic) have decreased electromyographic responses and increased electromechanical delays following spinal manipulation of the lumbar region compared to a corresponding asymptomatic group [41]. However, manipulative forces were not directly measured in that study, but were estimated based on a prediction algorithm derived in a different study. Therefore, the precise onset of the treatment thrust (needed to calculate the electromechanical delays) is associated with some uncertainty, and the absolute forces applied during the manipulations cannot be compared with confidence between the asymptomatic and symptomatic groups. For example, it could be that the different electromyographic responses were related to systematic differences between the treatment forces applied to the two experimental groups.

In contrast to this, our study directly measured the forces applied during manipulation and thus the exact timing of thrust onset. Our results support the hypothesis that differences in treatment forces influence electromyographic responses and electromechanical delays. Specifically, our results show that manipulations with a greater rate of force application and more forceful thrusts result in greater electromyographic responses with less electromechanical delay. This was observed in manipulations delivered to the lower cervical and upper thoracic spine in the symptomatic group and for the thoracic region in the asymptomatic group. We also observed this pattern with manipulations delivered to the upper cervical spine in both groups and in the lower cervical spine in the asymptomatic group, but the results did not reach statistical significance, likely due to an insufficient number of comparisons in these regions. Interestingly, there was a greater absolute force delivered at both the upper and lower cervical spines for the asymptomatic population. This systematic difference between the two groups is curious, especially considering that a single practitioner delivered all manipulations. Typically, manipulations delivered by a practitioner to a spinal region (e.g. neck) are consistent [29] while thrusts delivered to the same region by different clinicians are highly variable [42]. One possible reason for this difference between populations could be previous experience with spinal manipulation. Specifically, in the asymptomatic group, the participants were naïve to spinal manipulation prior to their involvement in the study while the symptomatic group was recruited from the existing patient base of the chiropractor. Thus, factors such as participant familiarity, anxiety related to cervical spine manipulation and the practitioner-patient relationship may have affected the level of force applied by the chiropractor.

Many clinicians judge the success of an HVLA manipulation by the presence of a cracking, clicking or popping sound – commonly known among manual therapists as cavitation [23–25]. The first study on cavitation associated with HVLA manipulation was conducted on metacarpophalangeal joints. It has been suggested that an increase in the joint space and an associated increase in joint volume with the HVLA manipulation caused the collapse of intra-articular gas bubbles which were responsible for the sound of cavitation [43]. However, more recent studies using advanced imaging to study both metacarpal joints in the hands and zygapophyseal joint spaces in the cervical spine do not support this earlier hypothesis. Rather, it was reported there was no evidence of gas within the joint space nor an increase in joint space immediately post-manipulation. Further to this, no vacuum phenomena were seen [44, 45]. Thus, the mechanisms underlying spinal joint cavitation are still unknown [23] and they may differ from those observed in metacarpophalangeal joints. Despite reports that cavitation elicited by HVLA spinal manipulation causes reflex responses [28] and that spinal manipulations delivered to the low back that elicited cavitation were associated with decreased sensitivity of muscle spindles of paraspinal erector spinae muscles [46], the current results cannot support this hypothesis. Rather, the current results support the literature arguing that cavitation elicited by HVLA spinal manipulation does not by itself cause reflex responses [29, 30]. Irrespective of this debate, many practitioners will immediately deliver a second thrust in a clinical situation if cavitation is not achieved with the first thrust [23, 26, 27].

### Limitations

As reflex responses associated with spinal manipulation typically occur within 500 ms of the onset of thrust, it is possible that the electromyographic response associated with the second thrust may not be independent of that occurring with the first thrust. Indeed, the delivery of two thrusts within a short period of time made it impossible to define a reliable preload force prior to the second thrust. This is a salient point as modification of preload forces are another force-time parameter important for altering electromyographic responses associated with manipulation [7, 9, 47]. However, inspection of the data suggests that the electromyographic responses elicited by the first thrust returned to baseline prior to the onset of the second thrust. Therefore, there was no direct summation of remnant electromyographic signal from the first thrust with the second thrust. Thus, we believe that it is likely that the same/similar differences would be observed if the two thrusts were measured independently. But there is the distinct possibility that even though the electromyographic signal of the first thrust had subsided prior to the second thrust, the reflex system may have been primed differently for the second compared to the first thrust and thus may have affected the electromyographic response of the second thrust. However, independent of what the exact mechanisms were for the increased reflex responses in the second compared to the first thrust, they would represent what happens in a clinical situation, and potentially might affect treatment outcomes. Further investigation into differences between preload forces and neural responses and the timing of manipulations that are delivered following one another in quick succession should be conducted.

It is possible that the electromyographic responses recorded in this study were inconsistent due to a number of factors. Firstly, variation in electrode placement between subjects may have occurred, resulting in the recording of electromyographic responses from different parts of the same muscle between participants. However, all possible care was taken to ensure that electrode placement was consistent between participants despite differences in body size, shape and anatomy. Furthermore, surface electromyographic is a reliable [48] and commonly used instrument for the measurement of electromyographic response to spinal manipulation [10, 12–14]. Secondly, it is possible that physical differences (e.g., size, weight, somatotype) between participants may have affected the force-time parameters of manipulation such as the line of drive, level of force applied and speed of the thrust. These differences could feasibly have changed the anatomy affected by the thrust and thus the electromyographic responses associated with manipulation. While we attempted to reduce this variability by using the same chiropractor with > 30 years’ experience delivering manual HVLA spinal manipulations in both studies, there were differences between the peak forces, rate of force application and electromyographic delays between the asymptomatic and symptomatic groups in both the first thrust and the second thrust. However, this variability is impossible to control when delivering a manual HVLA spinal manipulation to a human. Furthermore, as we compared responses between two thrusts delivered to the same participant, the effect of size or body type differences would be accounted for in the study design. Further, in some muscles (e.g., left latissimus dorsi, asymptomatic cohort), the electromyographic responses were very small and thus, a small absolute difference between two thrusts would become artificially large when the second thrust was normalized to the first. Thus, we recommend the reader exercises caution when interpreting the percentage difference between the two thrusts.

It is also possible that design limitations of the pressure pad used to record force-time parameters may have influenced our results. Specifically, the pad can only measure forces perpendicular to its surface. Any shear forces are not measured thus the reported forces tend to underestimate the actual forces applied by the chiropractor. Further to this, the pad is flexible and conforms to both the participant’s anatomy (spine and surrounding soft tissues) and the clinician’s hand. Bending of a pressure sensor would give a force that is not applied. However, the pressure pad is constructed with small elements that are spaced apart, and the spacing allows for great bending of the pad without bending of the sensors in the pad. Therefore, we do not believe that bending of sensors caused artifactual forces, and because of the repeated nature of the comparisons with matched contact positions, such artifacts would be expected to be the same for a given comparison pair, thus not affecting differences observed between the first and second thrust. The space between the sensors should not affect the forces measured, as according to Newton’s laws action is equal to reaction, and the empty space between sensors cannot support forces, so all forces applied by the chiropractor are measured by the contact sensors. Finally, we collected force data at a sampling rate of 200 Hz, which is reported to be adequate to describe HVLA manipulative thrusts [49]. Despite this, it is possible the peak forces may be somewhat underestimated. However, we think this underestimation would be relatively small, and it would be a systematic error that would not affect the relative differences between the 1st and 2nd thrust measurements. Nevertheless, the highest rate of force application we measured was 3710 N/s. Sampling at 200 Hz means that we get a force sample every 5 ms. In the worst-case scenario, our data points measured would be as far away as possible from where the actual peak force occurred, which would be exactly between two measured points (or 2.5 ms from the actual force peak). If so, and at the highest rate of 3710 N/s, we would potentially miss the peak force by 3710 N/s · 0.0025 s = 9.3 N. The mean peak forces for these measurements with the highest rate of force applications were 573 N (Table 2 in the manuscript). Therefore, an underestimation of 9.3 N would correspond to an error of about 9.3/573 = 0.016 or about 1.6% of the actual value. Since the rate of force application is always highest somewhere in the middle of the manipulative thrust, and the rate towards the peak force is much smaller (it becomes zero at the peak force), we can safely say that the sampling rate would cause a maximal error in peak force calculation that is less than 1%. Further, this potential offset of treatment forces does not influence the timing of events occurring during the manipulation (e.g., onset of thrust, peak force), which is what were used to analyze the electromyographic responses.

Additionally, the order of the manipulations was non-random – each participant underwent manipulation from C1-T4 in the same order and on the same side. Thus, it is possible that there may have been an order effect present. Specifically, there may have been both ascending and/or descending effects from both the spinal cord and/or brain. However, if this was the case, it is reasonable to assume, although not proven, that it would have affected the symptomatic and asymptomatic population in a similar manner. Regarding a possible order effect between the first and second thrusts, it has been reported that there is an attenuation of the H-reflex response with cervical spine manipulation [50], suggesting that the electromyographic responses associated with Ia muscle spindle reflexes might be depressed for the second compared to the first thrust. We observed the opposite of what a depressed H-reflex response would suggest: an increased electromyographic responses associated with the second thrust (the onset of which occurred after the electromyographic response returned to baseline following the first thrust). However, since we expect that the reflex responses measured in our study arise from a multitude of sources, and not only the Ia muscle spindle pathway, and since there are no reports on sensitivities of other reflex pathways following cervical spinal manipulation, it is hard to speculate if there was an order effect or if the increased reflex responses in the second thrust were a reflection of the altered mechanics compared to the first thrust. Independent of the answer to this question, it appears that a second thrust given in a clinically relevant situation enhances the reflex response and may have implications for the treatment outcomes.

## Conclusion

When a second spinal manipulation was delivered because there was not audible cavitation with the first thrust, greater treatment forces and faster thrust rates were associated with the second thrust. Furthermore, greater peak electromyographic responses were seen following the second thrusts. This is the first systematic analysis of the differences in force-time parameters and electromyographic characteristics following manual HVLA spinal manipulations delivered following one another in quick succession by a clinician, and as such, further investigation into this relationship is required. Further, the relationship between force-time parameters and electromyographic responses in muscles in addition to the short-term and long-term clinical outcomes of cervical and upper thoracic spinal manipulation are urgently needed.

## Data Availability

The datasets used and/or analyzed during the current study are available from the corresponding author on reasonable request.

## Abbreviations

HVLA: High velocity, low amplitude
C: Cervical vertebrae
T: Thoracic vertebrae
RMS: Root-mean-square
